# 1,5-Bis(1-phenyl­ethyl­idene)thio­carbono­hydrazide

**DOI:** 10.1107/S1600536811018320

**Published:** 2011-05-25

**Authors:** Lei Feng, Haiwei Ji, Renliang Wang, Haiyan Ge, Li Li

**Affiliations:** a Atherosclerosis Institute, Taishan Medical University, 271000 Taian, People’s Republic of China; b College of Chemistry and Chemical Engineering, Taishan Medical University, 271016 Taian, People’s Republic of China

## Abstract

The title mol­ecule, C_17_H_18_N_4_S, is not planar, as indicated by the dihedral angle of 27.24 (9)° between the two benzene rings. In the crystal, inter­molecular N—H⋯S hydrogen bonds link pairs of mol­ecules into inversion dimers.

## Related literature

For the biological activity and catalytic abilities of Schiff base derivatives and complexes, see: Loncle *et al.* (2004 ▶); Camp *et al.* (2010 ▶). For a related structure, see: Meyers *et al.* (1995 ▶).
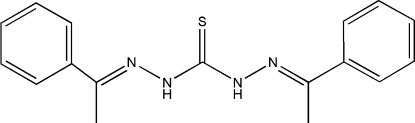


## Experimental

### 

#### Crystal data

C_17_H_18_N_4_S
*M* *_r_* = 310.41
Triclinic, 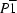

*a* = 7.5947 (15) Å
*b* = 8.8202 (18) Å
*c* = 13.084 (3) Å
α = 76.62 (3)°
β = 76.39 (3)°
γ = 82.35 (3)°
*V* = 825.8 (3) Å^3^
*Z* = 2
Mo *K*α radiation
μ = 0.20 mm^−1^
*T* = 293 K
0.20 × 0.16 × 0.12 mm

#### Data collection

Bruker SMART APEX CCD area-detector diffractometer
Absorption correction: multi-scan (*SADABS*; Sheldrick, 1996 ▶) *T*_min_ = 0.962, *T*_max_ = 0.977
4272 measured reflections
2876 independent reflections
2033 reflections with *I* > 2σ(*I*)
*R*_int_ = 0.022

#### Refinement

*R*[*F* ^2^ > 2σ(*F* ^2^)] = 0.047
*wR*(*F* ^2^) = 0.146
*S* = 1.02
2876 reflections
202 parameters
1 restraint
H-atom parameters constrained
Δρ_max_ = 0.24 e Å^−3^
Δρ_min_ = −0.19 e Å^−3^

### 

Data collection: *SMART* (Bruker, 2007 ▶); cell refinement: *SAINT* (Bruker, 2007 ▶); data reduction: *SAINT*; program(s) used to solve structure: *SHELXS97* (Sheldrick, 2008 ▶); program(s) used to refine structure: *SHELXL97* (Sheldrick, 2008 ▶); molecular graphics: *SHELXTL* (Sheldrick, 2008 ▶); software used to prepare material for publication: *SHELXTL*.

## Supplementary Material

Crystal structure: contains datablocks I, global. DOI: 10.1107/S1600536811018320/vm2088sup1.cif

Structure factors: contains datablocks I. DOI: 10.1107/S1600536811018320/vm2088Isup2.hkl

Supplementary material file. DOI: 10.1107/S1600536811018320/vm2088Isup3.cml

Additional supplementary materials:  crystallographic information; 3D view; checkCIF report

## Figures and Tables

**Table 1:** Hydrogen-bond geometry (Å, °)

*D*—H⋯*A*	*D*—H	H⋯*A*	*D*⋯*A*	*D*—H⋯*A*
N3—H3⋯S1^i^	0.86	2.67	3.515 (2)	169

Symmetry code: (i) 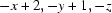.

**Fig. 1.**
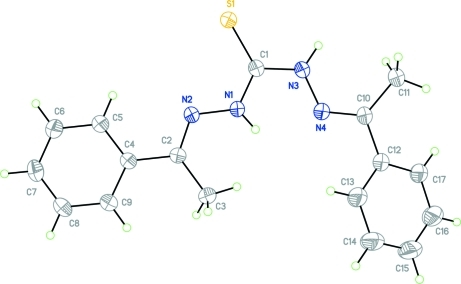
View of (I) showing the atomic numbering and 30% probability displacement ellipsoids.

**Fig. 2.**
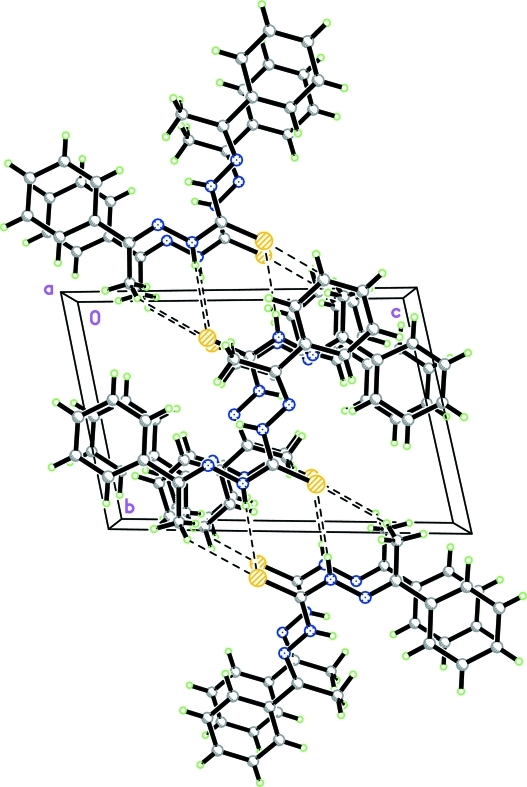
View along a-axis of the packing of (I). N—H···S interactions are represented by dashed lines.

## supplementary crystallographic information

### Comment

Much interest has recently been paid to the design of schiff base derivatives and complexes, owing to their wide range of biological activities and catalytical abilities (Loncle *et al.*, 2004; Camp *et al.*, 2010). We have synthesized title compound, (I), and report here its crystal structure (Fig. 1). The bond lengths and angles are normal and correspond to those observed in bis(3-fluorophenylmethine)carbonohydrazide (Meyers *et al.*, 1995).  The planes of atoms N2/N1/C1 and benzene ring C4-C9 form a dihedral angle of 2.62 (23)°, indicating that this part of the molecule is nearly planar. However, benzene rings C4-C9 and C12-C17 form a dihedral angle of 27.24 (9)°. Intermolecular N—H···S hydrogen bonds link two molecules into one dimer (Table 1, Fig. 2).

### Experimental

Acetophenone (10.0 mmol) and thiocarbohydrazide (5.0 mmol) were mixed in 50 ml flash under sovlent-free condtions After stirring 3 h at 373 K, the resulting mixture was cooled to room temperature, and recrystalized from ethanol, and afforded the title compound as a crystalline solid.

### Refinement

All H atoms were placed in geometrically idealized positions (N—H 0.86 and C—H 0.93–0.96 Å) and treated as riding on their parent atoms, with *U*_iso_(H) = 1.2–1.5*U*_eq_(C,*N*).

### Crystal data

**Table:**

C_17_H_18_N_4_S	*Z* = 2
*M**_r_* = 310.41	*F*(000) = 328
Triclinic, *P*1	*D*_x_ = 1.248 Mg m^−^^3^
Hall symbol: -P 1	Mo *K*α radiation, λ = 0.71073 Å
*a* = 7.5947 (15) Å	Cell parameters from 1351 reflections
*b* = 8.8202 (18) Å	θ = 2.6–25.1°
*c* = 13.084 (3) Å	µ = 0.20 mm^−^^1^
α = 76.62 (3)°	*T* = 293 K
β = 76.39 (3)°	Block, colourless
γ = 82.35 (3)°	0.20 × 0.16 × 0.12 mm
*V* = 825.8 (3) Å^3^	

### Data collection

**Table:**

Bruker SMART APEX CCD area-detector diffractometer	2876 independent reflections
Radiation source: fine-focus sealed tube	2033 reflections with *I* > 2σ(*I*)
graphite	*R*_int_ = 0.022
φ and ω scans	θ_max_ = 25.0°, θ_min_ = 2.6°
Absorption correction: multi-scan (*SADABS*; Sheldrick, 1996)	*h* = −8→9
*T*_min_ = 0.962, *T*_max_ = 0.977	*k* = −10→9
4272 measured reflections	*l* = −15→15

### Refinement

**Table:**

Refinement on *F*^2^	Secondary atom site location: difference Fourier map
Least-squares matrix: full	Hydrogen site location: inferred from neighbouring sites
*R*[*F*^2^ > 2σ(*F*^2^)] = 0.047	H-atom parameters constrained
*wR*(*F*^2^) = 0.146	*w* = 1/[σ^2^(*F*_o_^2^) + (0.0718*P*)^2^ + 0.2086*P*] where *P* = (*F*_o_^2^ + 2*F*_c_^2^)/3
*S* = 1.02	(Δ/σ)_max_ = 0.001
2876 reflections	Δρ_max_ = 0.24 e Å^−^^3^
202 parameters	Δρ_min_ = −0.19 e Å^−^^3^
1 restraint	Extinction correction: *SHELXL97* (Sheldrick, 2008), Fc^*^=kFc[1+0.001xFc^2^λ^3^/sin(2θ)]^-1/4^
Primary atom site location: structure-invariant direct methods	Extinction coefficient: 0.015 (4)

### Special details

**Table:**

Geometry. All e.s.d.'s (except the e.s.d. in the dihedral angle between two l.s. planes) are estimated using the full covariance matrix. The cell e.s.d.'s are taken into account individually in the estimation of e.s.d.'s in distances, angles and torsion angles; correlations between e.s.d.'s in cell parameters are only used when they are defined by crystal symmetry. An approximate (isotropic) treatment of cell e.s.d.'s is used for estimating e.s.d.'s involving l.s. planes.
Refinement. Refinement of *F*^2^ against ALL reflections. The weighted *R*-factor *wR* and goodness of fit *S* are based on *F*^2^, conventional *R*-factors *R* are based on *F*, with *F* set to zero for negative *F*^2^. The threshold expression of *F*^2^ > σ(*F*^2^) is used only for calculating *R*-factors(gt) *etc*. and is not relevant to the choice of reflections for refinement. *R*-factors based on *F*^2^ are statistically about twice as large as those based on *F*, and *R*- factors based on ALL data will be even larger.

### Fractional atomic coordinates and isotropic or equivalent isotropic displacement parameters (Å^2^)

**Table:**

	*x*	*y*	*z*	*U*_iso_*/*U*_eq_	
S1	0.91194 (11)	0.69768 (7)	−0.10817 (5)	0.0688 (3)	
N1	0.7946 (3)	0.9200 (2)	0.00704 (14)	0.0560 (6)	
H1	0.7779	0.9500	0.0669	0.067*	
N2	0.7386 (3)	1.0205 (2)	−0.07909 (14)	0.0521 (5)	
N3	0.9241 (3)	0.6971 (2)	0.09311 (14)	0.0566 (6)	
H3	0.9787	0.6046	0.0982	0.068*	
N4	0.8830 (3)	0.7712 (2)	0.17832 (15)	0.0520 (5)	
C1	0.8745 (3)	0.7765 (3)	−0.00005 (18)	0.0524 (6)	
C2	0.6582 (3)	1.1530 (3)	−0.06107 (18)	0.0498 (6)	
C3	0.6205 (4)	1.2028 (3)	0.0448 (2)	0.0677 (8)	
H3A	0.6098	1.1120	0.1022	0.102*	
H3B	0.5091	1.2689	0.0525	0.102*	
H3C	0.7185	1.2592	0.0474	0.102*	
C4	0.6017 (3)	1.2612 (2)	−0.15566 (18)	0.0514 (6)	
C5	0.6339 (4)	1.2169 (3)	−0.25393 (19)	0.0617 (7)	
H5	0.6893	1.1180	−0.2604	0.074*	
C6	0.5846 (4)	1.3179 (3)	−0.3422 (2)	0.0701 (8)	
H6	0.6078	1.2867	−0.4076	0.084*	
C7	0.5015 (4)	1.4645 (3)	−0.3343 (2)	0.0704 (8)	
H7	0.4694	1.5324	−0.3942	0.085*	
C8	0.4666 (4)	1.5095 (3)	−0.2381 (2)	0.0748 (8)	
H8	0.4092	1.6080	−0.2321	0.090*	
C9	0.5164 (4)	1.4089 (3)	−0.1491 (2)	0.0661 (8)	
H9	0.4921	1.4411	−0.0839	0.079*	
C10	0.9131 (3)	0.6990 (3)	0.27089 (18)	0.0516 (6)	
C11	0.9913 (4)	0.5327 (3)	0.2956 (2)	0.0708 (8)	
H11A	0.9160	0.4657	0.2797	0.106*	
H11B	0.9961	0.5032	0.3703	0.106*	
H11C	1.1118	0.5228	0.2525	0.106*	
C12	0.8641 (3)	0.7926 (3)	0.35463 (18)	0.0528 (6)	
C13	0.8581 (5)	0.9550 (3)	0.3268 (2)	0.0790 (9)	
H13	0.8894	1.0049	0.2550	0.095*	
C14	0.8063 (7)	1.0415 (4)	0.4049 (3)	0.1186 (16)	
H14	0.8012	1.1501	0.3855	0.142*	
C15	0.7621 (7)	0.9703 (4)	0.5109 (3)	0.1202 (16)	
H15	0.7270	1.0304	0.5631	0.144*	
C16	0.7691 (5)	0.8118 (4)	0.5402 (2)	0.0960 (12)	
H16	0.7384	0.7633	0.6124	0.115*	
C17	0.8217 (4)	0.7239 (3)	0.4628 (2)	0.0696 (8)	
H17	0.8291	0.6154	0.4834	0.084*	

### Atomic displacement parameters (Å^2^)

**Table:**

	*U*^11^	*U*^22^	*U*^33^	*U*^12^	*U*^13^	*U*^23^
S1	0.1111 (7)	0.0488 (4)	0.0460 (4)	0.0237 (4)	−0.0270 (4)	−0.0155 (3)
N1	0.0858 (16)	0.0416 (10)	0.0403 (10)	0.0161 (10)	−0.0229 (10)	−0.0105 (8)
N2	0.0758 (14)	0.0391 (10)	0.0407 (10)	0.0090 (9)	−0.0189 (9)	−0.0079 (8)
N3	0.0823 (15)	0.0436 (11)	0.0424 (11)	0.0156 (10)	−0.0214 (10)	−0.0092 (9)
N4	0.0683 (14)	0.0459 (11)	0.0418 (10)	0.0067 (9)	−0.0171 (9)	−0.0102 (8)
C1	0.0691 (17)	0.0430 (12)	0.0427 (13)	0.0073 (11)	−0.0156 (11)	−0.0071 (10)
C2	0.0654 (16)	0.0407 (12)	0.0446 (13)	0.0064 (11)	−0.0165 (11)	−0.0128 (10)
C3	0.097 (2)	0.0580 (15)	0.0537 (15)	0.0202 (14)	−0.0303 (15)	−0.0234 (12)
C4	0.0660 (16)	0.0410 (12)	0.0474 (13)	0.0063 (11)	−0.0176 (12)	−0.0103 (10)
C5	0.086 (2)	0.0492 (14)	0.0503 (14)	0.0137 (13)	−0.0234 (13)	−0.0130 (11)
C6	0.096 (2)	0.0658 (17)	0.0490 (14)	0.0156 (15)	−0.0264 (14)	−0.0144 (13)
C7	0.086 (2)	0.0609 (16)	0.0582 (17)	0.0102 (14)	−0.0271 (15)	0.0019 (13)
C8	0.103 (2)	0.0476 (14)	0.0700 (18)	0.0218 (15)	−0.0289 (16)	−0.0092 (13)
C9	0.094 (2)	0.0482 (14)	0.0533 (15)	0.0171 (13)	−0.0203 (14)	−0.0140 (12)
C10	0.0650 (16)	0.0453 (13)	0.0444 (13)	0.0039 (11)	−0.0194 (11)	−0.0058 (10)
C11	0.101 (2)	0.0559 (15)	0.0552 (15)	0.0207 (15)	−0.0320 (15)	−0.0116 (12)
C12	0.0649 (16)	0.0496 (13)	0.0446 (13)	0.0031 (11)	−0.0183 (11)	−0.0088 (11)
C13	0.132 (3)	0.0535 (16)	0.0543 (16)	0.0003 (16)	−0.0293 (17)	−0.0106 (13)
C14	0.230 (5)	0.0540 (18)	0.078 (2)	0.015 (2)	−0.049 (3)	−0.0248 (17)
C15	0.219 (5)	0.078 (2)	0.065 (2)	0.028 (3)	−0.036 (3)	−0.0342 (18)
C16	0.156 (3)	0.080 (2)	0.0468 (16)	0.014 (2)	−0.0207 (19)	−0.0171 (15)
C17	0.104 (2)	0.0558 (15)	0.0473 (15)	0.0050 (15)	−0.0223 (14)	−0.0075 (12)

### Geometric parameters (Å, °)

**Table:**

S1—C1	1.667 (2)	C7—H7	0.9300
N1—C1	1.343 (3)	C8—C9	1.388 (3)
N1—N2	1.378 (2)	C8—H8	0.9300
N1—H1	0.8600	C9—H9	0.9300
N2—C2	1.290 (3)	C10—C12	1.471 (3)
N3—C1	1.367 (3)	C10—C11	1.499 (3)
N3—N4	1.374 (3)	C11—H11A	0.9600
N3—H3	0.8600	C11—H11B	0.9600
N4—C10	1.288 (3)	C11—H11C	0.9600
C2—C4	1.490 (3)	C12—C17	1.383 (3)
C2—C3	1.502 (3)	C12—C13	1.392 (3)
C3—H3A	0.9600	C13—C14	1.368 (4)
C3—H3B	0.9600	C13—H13	0.9300
C3—H3C	0.9600	C14—C15	1.366 (5)
C4—C5	1.387 (3)	C14—H14	0.9300
C4—C9	1.388 (3)	C15—C16	1.358 (5)
C5—C6	1.380 (3)	C15—H15	0.9300
C5—H5	0.9300	C16—C17	1.370 (4)
C6—C7	1.376 (4)	C16—H16	0.9300
C6—H6	0.9300	C17—H17	0.9300
C7—C8	1.363 (4)		
			
C1—N1—N2	121.73 (18)	C7—C8—H8	119.9
C1—N1—H1	119.1	C9—C8—H8	119.9
N2—N1—H1	119.1	C8—C9—C4	121.1 (2)
C2—N2—N1	115.81 (18)	C8—C9—H9	119.5
C1—N3—N4	117.21 (18)	C4—C9—H9	119.5
C1—N3—H3	121.4	N4—C10—C12	114.8 (2)
N4—N3—H3	121.4	N4—C10—C11	124.5 (2)
C10—N4—N3	120.56 (19)	C12—C10—C11	120.7 (2)
N1—C1—N3	112.62 (19)	C10—C11—H11A	109.5
N1—C1—S1	125.52 (17)	C10—C11—H11B	109.5
N3—C1—S1	121.86 (17)	H11A—C11—H11B	109.5
N2—C2—C4	114.99 (19)	C10—C11—H11C	109.5
N2—C2—C3	125.2 (2)	H11A—C11—H11C	109.5
C4—C2—C3	119.78 (19)	H11B—C11—H11C	109.5
C2—C3—H3A	109.5	C17—C12—C13	117.6 (2)
C2—C3—H3B	109.5	C17—C12—C10	121.9 (2)
H3A—C3—H3B	109.5	C13—C12—C10	120.5 (2)
C2—C3—H3C	109.5	C14—C13—C12	120.2 (3)
H3A—C3—H3C	109.5	C14—C13—H13	119.9
H3B—C3—H3C	109.5	C12—C13—H13	119.9
C5—C4—C9	117.8 (2)	C15—C14—C13	120.7 (3)
C5—C4—C2	120.7 (2)	C15—C14—H14	119.6
C9—C4—C2	121.6 (2)	C13—C14—H14	119.6
C6—C5—C4	120.8 (2)	C16—C15—C14	120.2 (3)
C6—C5—H5	119.6	C16—C15—H15	119.9
C4—C5—H5	119.6	C14—C15—H15	119.9
C7—C6—C5	120.6 (2)	C15—C16—C17	119.6 (3)
C7—C6—H6	119.7	C15—C16—H16	120.2
C5—C6—H6	119.7	C17—C16—H16	120.2
C8—C7—C6	119.5 (2)	C16—C17—C12	121.6 (3)
C8—C7—H7	120.2	C16—C17—H17	119.2
C6—C7—H7	120.2	C12—C17—H17	119.2
C7—C8—C9	120.2 (2)		
			
C1—N1—N2—C2	177.7 (2)	C7—C8—C9—C4	−0.2 (5)
C1—N3—N4—C10	174.7 (2)	C5—C4—C9—C8	−0.6 (4)
N2—N1—C1—N3	178.3 (2)	C2—C4—C9—C8	179.1 (3)
N2—N1—C1—S1	−1.9 (4)	N3—N4—C10—C12	179.5 (2)
N4—N3—C1—N1	1.4 (3)	N3—N4—C10—C11	−0.6 (4)
N4—N3—C1—S1	−178.43 (18)	N4—C10—C12—C17	155.1 (3)
N1—N2—C2—C4	179.0 (2)	C11—C10—C12—C17	−24.8 (4)
N1—N2—C2—C3	−0.8 (4)	N4—C10—C12—C13	−24.6 (4)
N2—C2—C4—C5	0.8 (4)	C11—C10—C12—C13	155.6 (3)
C3—C2—C4—C5	−179.5 (2)	C17—C12—C13—C14	−1.9 (5)
N2—C2—C4—C9	−178.9 (3)	C10—C12—C13—C14	177.7 (3)
C3—C2—C4—C9	0.8 (4)	C12—C13—C14—C15	0.8 (6)
C9—C4—C5—C6	0.9 (4)	C13—C14—C15—C16	0.0 (7)
C2—C4—C5—C6	−178.8 (3)	C14—C15—C16—C17	0.3 (7)
C4—C5—C6—C7	−0.4 (5)	C15—C16—C17—C12	−1.5 (6)
C5—C6—C7—C8	−0.5 (5)	C13—C12—C17—C16	2.3 (5)
C6—C7—C8—C9	0.7 (5)	C10—C12—C17—C16	−177.3 (3)

### Hydrogen-bond geometry (Å, °)

**Table:**

*D*—H···*A*	*D*—H	H···*A*	*D*···*A*	*D*—H···*A*
N3—H3···S1^i^	0.86	2.67	3.515 (2)	169

Symmetry codes: (i) −*x*+2, −*y*+1, −*z*.
